# Polish Adaptation of the Pregnancy-Related Anxiety Questionnaire—Revised 2 for All Pregnant Women

**DOI:** 10.3390/healthcare9070917

**Published:** 2021-07-20

**Authors:** Anna Michalik, Lucyna Wójcicka, Agata Zdun-Ryżewska, Agnieszka Czerwińska-Osipiak, Michał Krzemiński, Jolanta Olszewska, Dagmara Klasa-Mazurkiewicz, Anja C. Huizink

**Affiliations:** 1 Department of Obstetrical and Gynaecological Nursing, Medical University of Gdansk, 80-211 Gdansk, Poland; aniamichalik@gumed.edu.pl (A.M.); lucyna.wojcicka@gumed.edu.pl (L.W.); agnieszka.czerwinska-osipiak@gumed.edu.pl (A.C.-O.); jolanta.olszewska@gumed.edu.pl (J.O.); 2 Department of Quality of Life Research, Medical University of Gdansk, 80-210 Gdansk, Poland; azdun@gumed.edu.pl; 3 Institute of Applied Mathematics, Faculty of Applied Physics and Mathematics, Gdansk University of Technology, 80-233 Gdansk, Poland; mickrzem@pg.edu.pl; 4 Department of Gynaecology, Oncologic Gynaecology and Gynaecological Endocrinology, Medical University of Gdansk, 80-214 Gdansk, Poland; dagmara.klasa-mazurkiewicz@gumed.edu.pl; 5 Department of Clinical Developmental Psychology, Vrije Universiteit Amsterdam, Van der Boechorststraat 1, 1081 BT Amsterdam, The Netherlands

**Keywords:** childbirth, fear of childbirth, pregnancy-related anxiety

## Abstract

Pregnancy-related anxiety (PrA) is a specific type of anxiety characteristic of the perinatal period. PrA can affect pregnancy and birth. However, no validated tool exists to measure PrA in Polish obstetric practice. The aim of this study was to translate the Pregnancy-Related Anxiety Questionnaire—Revised 2 (PRAQ-R2) into Polish and to evaluate its reliability and factorial and construct validity. This study was conducted in Poland as an online questionnaire in April 2020 and included 175 healthy women. To validate the PRAQ-R2, we used standardized tools for the measurement of general anxiety: the modified Visual Analog Scale (VAS), the Ten-Item Personality Inventory (TIPI), and the Hospital Anxiety and Depression Scale (HADS). Scale reliability was assessed using Cronbach’s alpha. Concurrent validity was evaluated by calculating Spearman’s rho correlation coefficients. Statistical analyses were performed using R ver. 4.0.2. Values for comparative fit index >0.90, Tucker–Lewis index >0.90, and root mean square error of approximation <0.08 indicated acceptable model fit, confirming the reliability of the three-factor structure of the translation. The subscales and total scores had good consistency (α > 0.7), and convergent validity was demonstrated. The PRAQ-R2 as translated into Polish represents the first validated tool in Poland to measure PrA for all pregnant women.

## 1. Introduction

Pregnant women tend to display unique types and intensities of emotional responses, and recently, researchers who study perinatal care have emphasized the negative impacts associated with prenatal maternal anxiety on pregnancy and birth outcomes. The terms pregnancy-related anxiety (PrA) and pregnancy-specific anxiety (PSA) were introduced into clinical practice to highlight the distinct nature of anxiety experienced during pregnancy. These terms describe the specific types of anxiety that are experienced during the prenatal period associated with the woman’s own health, concern about her appearance, the health and development of the fetus, the course of the forthcoming delivery, and early parenthood [1,2,3,4]. The particular nature of PrA is important to note because symptoms of PrA differ from those associated with general anxiety. PrA can be distinctly associated with the birth weights of neonates, the gestational age at the moment of delivery, and mood disorders during the postpartum period [3,5,6].

Fear of childbirth (FOC) is a specific type of PrA [7,8,9], and 9–36% of all women experience severe FOC [4]. FOC has been well documented and is associated with the prolonged duration of active labor; increased use of pain relief during labor; higher rates of emergency cesarean births; and higher rates of obstetric interventions, including cesarean births and labor induction, performed without medical indications [10,11,12,13,14,15]. PrA has also been associated with negative personal experiences for pregnant women. Available reports have indicated that high PrA and FOC rates may be correlated with an increased risk of premature labor, low birth weights, and negative impacts on the neurological and behavioral development of neonates [6,16,17,18]. Moreover, a relationship has been noted between PrA and unhealthy behaviors during pregnancy (e.g., excessive weight gain during pregnancy) [7].

Differences in PrA scores have been identified between nulliparas and multiparas [8,10,19,20]. In multiparas, PrA and FOC are more often associated with emergency cesarean births, and instrumental labor is considered to be particularly traumatic for this population [14,21,22,23].

No standardized guidelines have been established for the diagnostic process of assessing PrA, and no standardized diagnostic criteria exist to guide the identification of PrA. While PrA occurs worldwide, the factors associated with the occurrence of PrA differ depending on individual and social factors and can vary across cultural and ethnic groups. Good obstetric practice requires the introduction of evidence-based recommendations for specific populations to assist in the identification and management of women who experience intense PrA and FOC [8,24,25]. The World Health Organization (WHO) recommended the introduction of psychoeducation for women with elevated PrA and FOC scores as a non-clinical recommendation for reducing the occurrence of medically unnecessary cesarean births [11]. Reduction of PrA is particularly relevant, because PrA is a stronger predictor of adverse pregnancy outcomes than general anxiety and depression [3,19,26].

The Pregnancy-Related Anxiety Questionnaire—Revised 2 (PRAQ-R2) is one of the specific tools that have been developed for the evaluation of PrA and has been validated for different cultures [1,27,28]. Multiple studies have confirmed the psychometric properties of the PRAQ-R2, and further adaptations have been developed as the popularity of this tool has increased [25,29,30,31,32,33,34].

The aim of this study was to translate the PRAQ-R2 and to evaluate its reliability and factorial and construct validity for a Polish population.

## 2. Materials and Methods

### 2.1. Study Design and Participants

This study was performed in April 2020 and included healthy women in their second or third trimesters of pregnancy. Each respondent was informed about the aim of the study and the planned method for the publication of results, and each woman voluntarily participated in the study after providing informed consent. On 11 March 2020, the WHO assessed the coronavirus disease 2019 (COVID-19) outbreak as a pandemic; therefore, we opted to perform this study using the CAWI (Computer-Assisted Web Interview) method. The questionnaire was made available on two Polish blogs dedicated to the topic of pregnancy, childbirth, and parenthood and on their associated social media sites. The link to this study was also distributed via online support groups dedicated to pregnant women. The questionnaire was made available with a link to an eligibility screener. The inclusion criteria (currently pregnant women, no existing indications for a cesarean birth (CB), and no history of psychiatric disorders) were met by 175 pregnant women who had completed 14 weeks of gestation (*n* = 95 nulliparous and *n* = 80 parous). The following exclusion criteria were applied: the existence of comorbidities, including oncologic diseases, mental disorders and documented episodes of depression; high-risk pregnancies (pregnancy-induced hypertension, diabetes, fetus diseases and malformations); pregnancies after assisted reproductive technologies; and pregnancies with medical indications for CB. According to the guidelines for reliable factor analysis [13,35], the size of the sample was considered to be “fair,” and the decision was made to attempt to translate the PRAQ-R2 into Polish. Women completed the PRAQ-R2 only during pregnancy. To verify the concurrent validity of the PRAQ-R2 questionnaire, other popular and well-documented and validated tools were also used, including the Hospital Anxiety and Depression Scale (HADS), the Ten-Item Personality Inventory (TIPI), and the modified Visual Analog Scale (VAS). Each participant was asked to fill in all the scales. There was no general time requirement to complete the entire survey. However, once a respondent exited a tool, they were unable to reopen it.

The protocol for the study was approved by the Independent Bioethics Committee for Scientific Research at the Medical University of Gdańsk.

### 2.2. Research Tools

A cross-sectional, descriptive questionnaire was administered to participants to obtain background characteristics. The form consisted of 20 questions related to demographic and obstetric characteristics, including age, education, place of residence (urban/rural), marital status, vocational status, economic condition, comorbidities during pregnancy (diagnosed by a physician), gestational week, parity, obstetric history, and participation in antenatal classes.

To confirm the representative value of the study sample, the authors referred to two large Polish population research projects: a study on perinatal care at maternity units in 2015, which was conducted by the state control unit; and a report monitoring maternity units and the medicalization of births in Poland, as of 2017, which was conducted by a non-governmental foundation focused on improving perinatal care in Poland. The participants included in the present study did not differ significantly from the respondents involved in these two studies in terms of sociodemographic characteristics [36,37].

#### 2.2.1. Hospital Anxiety and Depression Scale (HADS)

The HADS is a reliable tool used to measure the intensity of anxiety (statements concerning general nervousness, tension, and fear) and the occurrence of depressive symptoms (statements concerning anhedonia, reduced mood, sadness, and loss of interest) among a group of patients [15]. HADS has been successfully adapted for a Polish context and has been used as a screening tool for the diagnosis of psychological disorders (including anxiety) by various clinical groups [16].

#### 2.2.2. Ten-Item Personality Inventory (TIPI)

The TIPI is a short questionnaire that was developed based on the Big Five Personality Theory and measures the following features: extraversion (reserved or outgoing), emotional stability (sensitive or confident), agreeableness (challenging or friendly), conscientiousness (easy-going or organized), and openness to experience (cautious or curious) [17]. This questionnaire was adequately adapted for a Polish context and remains an extraordinarily popular tool for describing personality [18].

#### 2.2.3. Modified Visual Analog Scale (VAS)

The modified VAS is used to measure the level of anxiety. Participants were asked to subjectively evaluate their levels of anxiety on a scale of 0 to 10 points, where 0 indicated no anxiety and 10 referred to intense anxiety. Although the VAS was initially developed to measure pain intensity, these types of scales are currently used for a broad spectrum of applications, including the estimation of anxiety in medical practice and scientific research [38,39,40,41,42,43].

#### 2.2.4. Pregnancy-Related Anxiety Questionnaire—Revised 2

The first version of the PRAQ tool was developed by Van den Bergh (1990). The extensive version of the PRAQ questionnaire (34 items) was initially revised by Huizink et al. (2004) into a 10-item form [7]. However, the PRAQ-R was dedicated for use in nulliparas only. A second modification (PRAQ-R2), which universalized the tool for all pregnant women, regardless of parity, was later introduced by Huizink [27]. We obtained consent from the author of the PRAQ-R2 to translate the tool into Polish.

The PRAQ-R2 consists of 10 questions, which are grouped into three subscales: fear of giving birth (FoGB; items 1, 2, and 6); worries of bearing a physically or mentally handicapped child (WaHC; items 4, 9, 10, and 11); and concern about own appearance (CoA; items 3, 5, and 7). An additional item, normally used in the PRAQ-R (item 8: “I am anxious about the delivery because I have never experienced one before”), is dedicated to nulliparas only and is used to differentiate the scores obtained in nullipara and multipara groups. In the PRAQ-R2, this item has been rephrased into universal item 1 (“I am anxious about the delivery”). The total score (ranging from 10 to 50 points) and the sum of item scores that constitute each of the three subscales can be calculated. Higher scores are assumed to indicate increased PrA intensity. No clinical cutoff point has been defined for this questionnaire. Although this tool is a self-report measure, it may also be used during an interview with a participant.

The first phase of the PRAQ-R2 questionnaire adaptation included the translation of the questions into Polish. Four experts were involved in this process: two Polish academic teachers (obstetric field) who teach in English, and two linguists (native English speakers who speak Polish fluently). The translation was performed using a forward–backward technique, according to the guidelines developed for the cross-cultural adaptations of health-related measures [22]. Consequently, we developed the Polish version of the PRAQ-R2 to measure PrA (See Appendix Table A1 for all items used in the study).

Another aim of this study was to evaluate the reliability and accuracy of the tool used for PrA measurement, regardless of the respondents’ parity. The hypothesized model of the construct, which was built on the three-dimensional structure of the form, was based on theory and previous analytical research [21]. The fit of the data in a hypothesized measurement model was tested using confirmatory factor analysis (CFA), which is commonly used in social and psychological research [23]. For the PRAQ-R2, standardized factor loadings and item–total correlations were compared separately for groups of nulliparous and multiparous women. Subsequently, the obtained results were compiled into combined groups to compare the measurements obtained from both the PRAQ-R and PRAQ-R2. The fit of the CFA models was evaluated with the recommended statistical tests [24]. Good model fit indicates that the model is plausible [25].

### 2.3. Statistical Analyses

Continuous variables are expressed as the mean ± standard deviation (SD). Categorical (dichotomous) variables are expressed as frequencies (%). Groups of nulliparous and parous women were compared using the Wilcoxon rank-sum test, Fisher’s exact test, or chi-square test of independence, as appropriate.

To assess the factorial validity of the three-factor model of PRAQ-R2, as described by Huizink [21], CFA was employed. The fit of the CFA models was evaluated using the chi-square test, comparative fit index (CFI), Tucker–Lewis index (TLI)/non-normed fit index (NNFI), root mean square error of approximation (RMSEA), and a 90% confidence interval (CI) for RMSEA. The fit was considered acceptable when values were above 0.90 for CFI and TLI and below 0.08 for RMSEA [24]. An alpha value of 0.05 was considered significant.

To perform the reliability analysis/internal consistency determination of the information gathered by the PRAQ-R/PRAQ-R2, Cronbach’s alpha values were calculated [26]. Wherever PRAQ-R scores were considered for parous women, item 8 (“I am anxious about the delivery because I have never experienced one before”) was omitted.

Differences in mean item scores between nulliparous and parous groups were evaluated with the (non-parametric) Mann–Whitney U test. Correlations between PRAQ-R/R2 scores and other related measures (VAS, TIPI, and HADS) were evaluated using Spearman’s rho coefficient.

All statistical analyses were performed using R ver. 4.0.2. [27] The CFA was performed using “lavaan” [28], whereas all model plots were generated with “semPlot” [29].

## 3. Results

### 3.1. Descriptive Statistics

The mean age of participants was 30.0 ± 4.43 years. The majority of respondents had higher (minimum of a bachelor’s degree) (80.6%) and secondary (16.6%) education. Most respondents lived in cities (80.6%) and were employed and professionally active (84.6%). The mean gestational age of the study group was 28.3 ± 7.5 weeks. The mean number of pregnancies at the time of the study was 1.77. Detailed demographic and obstetric characteristics are presented in Table 1.

The groups of nulliparous and parous women differed significantly for particular PRAQ-R/R2 values (Table 2, *p* < 0.05), including items 2, 3, and 6 (items 2 and 6 from the FoGB subscale; item 3 from the CoA subscale). The nulliparas obtained higher scores for particular item values and consequently higher total scores for both the PRAQ-R and PRAQ-R2 scales. Item 8 of the PRAQ-R was not evaluated in the parous group (Table 3).

For nulliparous women, the CFA of the three-factor structure of the PRAQ-R2 showed a good fit (χ^2^(32) = 31.391; *p* > 0.05; CFI = 1.00; TLI = 1.00; RMSEA < 0.001; RMSEA 90% CI: 0.0–0.074).

For parous women, the CFA indicated an even better fit for the PRAQ-R2 (χ^2^(32) = 21.779; *p* > 0.05; CFI = 1.00; TLI = 1.00; RMSEA < 0.001; RMSEA 90% CI: 0.0–0.033).

**Figure 1:**
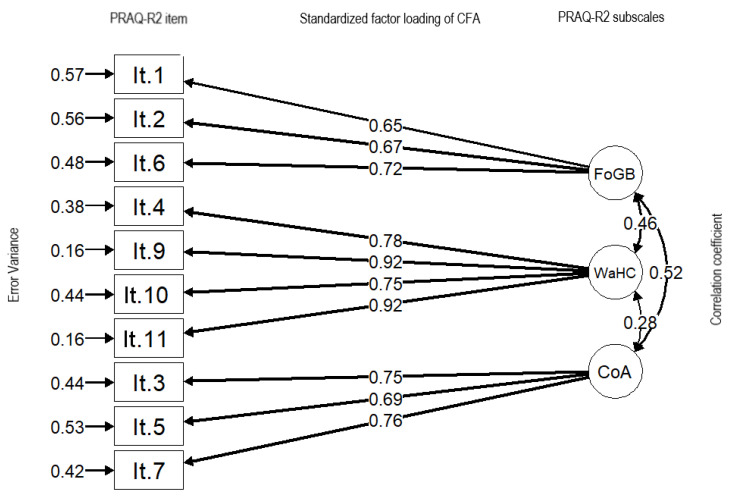
Standardized parameter estimates for the factor structure of the PRAQ-R2. FoGB: fear of giving birth; WaHC: worries about bearing a handicapped child; CoA: concern about one’s appearance.

The final standardized parameter estimates for the factor structure of the PRAQ-R2 are shown in Figure 1. All factor loadings for this model were significant (*p* < 0.05), and all items featured factor loadings greater than 0.65 based on their own latent factors.

### 3.2. Reliability

Internal consistency was assessed by calculating and evaluating Cronbach’s alpha coefficient for each subscale of the PRAQ-R2 in both nulli- and multiparous models and of the PRAQ-R/PRAQ-R2 in models for the combined groups. The validated models consisted of three items measuring the FoGB subscale and four items each measuring the WaHC and CoA scales. The impact of each item included in the model was assessed by computing Cronbach’s alpha when the respective item was deleted. None of the items included in the analysis showed alpha values greater than the final alpha value (compare Table 3 and Table 4). In every model, all three subscales showed high internal consistency, with Cronbach’s alpha coefficient values ranging from 0.68 to 0.94. The PRAQ-R2 and PRAQ-R models for the combined group showed similar internal consistency, with a slight advantage for the PRAQ-R2 over the PRAQ-R for the first subscale (FoGB), likely due to the extra item.

The relationship between anxiety and depression, as measured by HADS, and PRAQ-R/R2 scores confirmed positive, significant rho values, ranging from 0.17 to 0.38. Significant correlations were observed between every subscale of the tool (the strongest value was identified for the FoGB subscale) and the anxiety subscale of the HADS tool, which confirms the accuracy of the Polish adaptation of the PRAQ-R2. The comparison between the Polish adaptation of the PRAQ-R2 and the depression subscale of HADS was similarly satisfactory.

The personality questionnaire (TIPI) used in the present study allowed the PrA phenomenon to be correlated with the personality characteristics of the study participants. A negative and generally significant correlation (highest Rho of −0.37) was observed between the total and subscores of the PRAQ-R/R2 and the stability measure assessed by the TIPI, which indicated that increased neuroticism was associated with increased anxiety. Table 5 presents the complete matrix of correlations between the PRAQ-R/R2 and other anxiety measurement tools.

## 4. Discussion

The proper translation of the PRAQ-R2 for a Polish population is crucial to recognize a high level of PrA and to classify a pregnant woman into a high-risk group to provide her with specialized care. The conducted research shows that the PRAQ-R2 is an appropriately valid and reliable tool ready for use with all Polish pregnant women regardless of parity.

### 4.1. Reliability

The reliability of the PRAQ-R2 was evaluated with the Cronbach’s alpha internal consistency coefficient, item–total correlations, and correlation between the two forms of the PRAQ (PRAQ-R/PRAQ-R2) for both nulli- and multiparous models and in models for the combined groups. The internal consistency of our constructs measured by Cronbach’s alpha was high (over 0.7) for the total score and all three factors. The results are comparable between the PRAQ-R and PRAQ-R2 scales. Cronbach’s alpha for PRAQ-R was 0.77 in the nulliparous group, 0.86 in the parous group, and 0.81 for both groups combined. For the PRAQ-R2, these scores were marginally higher: 0.77 in the nulliparous group, 0.87 in the parous group, and 0.83 for the combined groups. All coefficients were also highly reliable (*p* = 0.001). These results are consistent with the results from the original PRAQ-R2 [21]. Specifically, in the original version of the questionnaire, Cronbach’s alpha internal consistency coefficient ranged from 0.71 to 0.82 in multiparous pregnant women and from 0.77 to 0.84 in nulliparous pregnant women. A suitable Cronbach’s alpha internal consistency coefficient should be as close as possible to a value of 1.

The overall correlation of each item with the questionnaire score is presented in Table 3 and Table 4. Corrected item–total correlations are relatively substantial (moderate to high); all values are greater than 0.4, which is above the recommended value for item selection (≥0.20). More precisely, values ranged from 0.41 to 0.73 in the nulliparous group and from 0.41 to 0.83 in the multiparous group (Table 3). For the combined groups, item–total correlation coefficients ranged from 0.42 to 0.75 (Table 4). The high correlation coefficients for each of the items shows that they are efficient and adequate in measuring intended behavior; they also are statistically significant (*p* = 0.001). As the last column of Table 4 (Cronbach’s alpha with item deleted) shows, all scores are less than 0.83. Hence, all 11 items were retained in further models. The current study results are close to the item–total correlation coefficient results shown in the original questionnaire [21]. The total correlation coefficients in the original questionnaire were 0.52–0.67 for the primiparous group and 0.47–0.72 for the multiparous group.

### 4.2. Validity

CFA was used to test the construct validity of the PRAQ-R2 adapted for Polish women. The analysis supported the three-factor scale structure, and the goodness-of-fit indices were used to assess the fit for the data (Figure 1). As with other analyses of psychometric properties [21], for the PRAQ-R2, standardized factor loadings and item–total correlations were compared separately for groups of nulliparous and multiparous women. Subsequently, the obtained results were compiled into combined groups to compare the measurements obtained from both the PRAQ-R and PRAQ-R2. Our results are comparable to the psychometric properties previously reported for the PRAQ-R2 [25,34].

The CFA of the three-factor structure of PRAQ-R and PRAQ-R2 showed good fit to all data regardless of parity. For combined groups, for PRAQ-R2, CFA indicated good fit, though slightly worse than that for PRAQ-R according to RMSEA. Negligibly worse fit has no effect on the obtained results, because all indicators indicate a very good match (Table 4). Therefore, the fit indices of the three-factor model for PRAQ-R2 showed room for improvement in the specification of the model, i.e., including correlated error terms, allowing items to load on more than one factor, or eliminating items, but the ease of use of the tool regardless of women’s parity outweighs the slight flaws of the model.

The construct validity of the PRAQ-R2 was verified by comparing this tool with other questionnaires and measures that are commonly used in the field of obstetrics and midwifery. The statistically significant correlations between the PRAQ-R2 and PRAQ-R scores and the anxiety measurements made using the modified VAS scale (the strongest value was identified for the FoGB subscale), the anxiety subscale of the HADS tool, and the measure of neuroticism (TIPI) support the adequate accuracy of the Polish translation of the PRAQ-R2, encouraging its further use. The comparison between the Polish version of the PRAQ-R2 and the depression subscale of HADS was similarly satisfactory. The present study confirmed the reported findings regarding the concurrent occurrence of anxiety and depression, which is frequently observed in psychiatric practice [33,44,45,46,47,48,49,50,51,52,53]. The correlation between anxiety and depression has been well documented and is associated with the prolonged duration of active labor; increased use of pain relief during labor; higher rates of emergency cesarean births; and higher rates of obstetric interventions, including cesarean births and labor induction, performed without medical indications [10,11,12,13,14,15].

The personality questionnaire (TIPI) used in the present study allowed PrA to be correlated with the personality characteristics of the study participants. A negative and generally significant correlation was observed between the total and subscores of the PRAQ-R/R2 and the emotional stability measure assessed by the TIPI, which indicated that increased neuroticism was associated with increased anxiety. High levels of neuroticism are associated with a tendency to be worried and experience stress, reactions that tend to be characterized by anxiety and tension. The correlation between high scores on the neuroticism scale and increased scores for various measures used to assess PrA is well documented [54,55].

Findings obtained from the study of Polish women are consistent with analysis of results from the original Dutch version of the questionnaire. We confirmed the assumed three-factor structure of the questionnaire with CFA. The Cronbach’s alpha internal consistency coefficient, item–total correlation, and correlation between the two forms of the PRAQ (PRAQ-R and PRAQ-R2) achieved a satisfactory level. The study results demonstrated that the Polish version of the PRAQ-R2 has a good fit with the original questionnaire and is a valid and reliable tool for use regardless of women’s parity.

The use of an easily accessible and understandable questionnaire may allow medical personnel to classify a pregnant woman into a high-risk group and provide her with specialized care, such as education targeted to affect the level of anxiety. This particular psychometric tool can be used for both research and clinical practice applications to measure and diagnose the anxiety level of pregnant women.

## Figures and Tables

**Table 1:** Demographic and obstetric characteristics of the study population.

Characteristic	Total *n* = 175	Primiparas *n* = 95 ^1^	Multiparas *n* = 80 ^1^	*p*-Value ^2^
Age (years)	30.02 ± 4.43	28.20 ± 4.01	32.17 ± 3.92	<0.001
Gestational week	28.34 ± 7.56	28.45 ± 8.18	28.21 ± 6.80	0.5
Number of pregnancies	1.77 ± 0.97	1.09 ± 0.44	2.58 ± 0.81	<0.001
Number of childbirths	0.57 ± 0.72	0.00 ± 0.00	1.25 ± 0.54	<0.001
Educational level				0.8
Vocational	5 (2.86%)	3 (3.16%)	2 (2.50%)	
High school	29 (16.57%)	14 (14.74%)	15 (18.75%)	
University	141 (80.57%)	78 (82.11%)	63 (78.75%)	
Place of residence				>0.9
Urban	141 (80.57%)	76 (80.00%)	65 (81.25%)	
Rural	34 (19.43%)	19 (20.00%)	15 (18.75%)	
Civil status				>0.9
Single	0 (0%)	0 (0%)	0 (0%)	
Married or cohabiting	175 (100%)	95 (100%)	80 (100%)	
Occupation				0.4
Active	148 (84.57%)	83 (87.37%)	65 (81.25%)	
Inactive	27 (15.43%)	12 (12.63%)	15 (18.75%)	
Financial situation				0.12
Very good	38 (21.71%)	16 (16.84%)	22 (27.50%)	
Good	131 (74.86%)	74 (77.89%)	57 (71.25%)	
Bad or very bad	6 (3.43%)	5 (5.26%)	1 (1.25%)	
Participation in antenatal classes	90 (51.43%)	42 (44.21%)	48 (60.00%)	0.054

^1^ Statistics presented: mean ± standard deviation or *n* (%). ^2^ Statistical tests performed: Wilcoxon rank-sum test; Fisher’s exact test; chi-square test of independence.

**Table 2:** Mean values of PRAQ-R2 items and significant differences between nulliparous and parous women.

Item Number	Total *n* = 175	Primiparas *n* = 95 ^1^	Multiparas *n* = 80 ^1^	*p*-Value ^2^
Item 1	4.01 ± 1.07	4.05 ± 1.00	3.95 ± 1.15	0.7
Item 2	3.55 ± 1.40	3.85 ± 1.27	3.20 ± 1.47	0.002
Item 3	2.30 ± 1.33	2.69 ± 1.39	1.84 ± 1.08	<0.001
Item 4	2.95 ± 1.21	3.06 ± 1.17	2.83 ± 1.26	0.2
Item 5	2.44 ± 1.42	2.53 ± 1.46	2.34 ± 1.37	0.4
Item 6	2.57 ± 1.52	2.89 ± 1.52	2.17 ± 1.44	<0.001
Item 7	2.32 ± 1.36	2.44 ± 1.37	2.17 ± 1.36	0.2
Item 8	4.06 ± 1.27	4.06 ± 1.27	–	
Item 9	2.72 ± 1.40	2.85 ± 1.43	2.56 ± 1.37	0.2
Item 10	2.40 ± 1.37	2.44 ± 1.35	2.35 ± 1.40	0.6
Item 11	2.52 ± 1.36	2.62 ± 1.35	2.40 ± 1.38	0.2

^1^ Statistics presented: mean ± standard deviation. ^2^ Statistical tests performed: Wilcoxon rank-sum test.

**Table 3:** Standardized factor loadings and item–total correlations for the PRAQ-R2 in primiparous and multiparous women (primiparous/multiparous).

PRAQ-R2 Item	Fear of Giving Birth (FoGB)	Worries about Bearing a Handicapped Child (WaHC)	Concern about Own Appearance (CoA)	Mean (SD)	Corrected Item–Total Correlations	Cronbach’s Alpha if Item Deleted
1	0.50/0.74			4.05 (1.00)/3.95 (1.15)	0.45/0.61	0.75/0.86
2	0.62/0.63			3.85 (1.27)/3.20 (1.47)	0.43/0.55	0.76/0.86
6	0.82/0.70			2.89 (1.52)/2.17 (1.44)	0.52/0.59	0.75/0.86
4		0.73/0.88		3.06 (1.17)/2.83 (1.26)	0.53/0.82	0.75/0.84
9		0.94/0.87		2.85 (1.43)/2.56 (1.37)	0.73/0.77	0.72/0.84
10		0.64/0.87		2.44 (1.35)/2.35 (1.40)	0.44/0.78	0.76/0.84
11		0.89/0.92		2.62 (1.35)/2.40 (1.38)	0.67/0.83	0.73/0.84
3			0.85/0.56	2.69 (1.39)/1.84 (1.08)	0.58/0.41	0.74/0.87
5			0.66/0.75	2.53 (1.46)/2.34 (1.37)	0.41/0.53	0.76/0.86
7			0.75/0.78	2.44 (1.37)/2.17 (1.36)	0.49/0.55	0.75/0.86
Cronbach’s alpha	0.70/0.73	0.88/0.94	0.80/0.74	Total 0.77/0.87		

PRAQ-R2: Pregnancy-Related Anxiety Questionnaire—Revised 2; SD: standard deviation.

**Table 4:** Factor loadings and item–total correlations for the PRAQ-R and the PRAQ-R2 in combined groups (PRAQ-R/PRAQ-R2).

PRAQ-R/R2 Item	Fear of Giving Birth (FoGB, Items 1, 2, 6)	Worries about Bearing a Handicapped Child (WaHC, Items 4, 9, 10, 11)	Concern about Own Appearance (CoA, Items 3, 5, 7)	Mean (SD)	Corrected Item–Total Correlations	Cronbach’s Alpha if Item Deleted
1	–/0.65			4.01 (1.07) *	–/0.53	–/0.82
2	0.61/0.67			3.55 (1.40)	0.43/0.51	0.81/0.82
6	0.89/0.72			2.57 (1.52)	0.57/0.57	0.80/0.81
4		0.78/0.78		2.95 (1.21)	0.68/0.68	0.79/0.80
9		0.91/0.92		2.72 (1.40)	0.75/0.75	0.78/0.80
10		0.75/0.75		2.40 (1.37)	0.61/0.61	0.80/0.81
11		0.93/0.92		2.52 (1.36)	0.76/0.75	0.78/0.80
3			0.76/0.75	2.30 (1.33)	0.54/0.52	0.80/0.82
5			0.68/0.69	2.44 (1.42)	0.49/0.47	0.81/0.82
7			0.75/0.76	2.32 (1.36)	0.53/0.52	0.80/0.82
Cronbach’s alpha	0.68/0.72	0.91/0.91	0.78/0.78	Total 0.82/0.83		

* Not applicable for PRAQ-R. PRAQ-R: Pregnancy-Related Anxiety Questionnaire—Revised; SD: standard deviation.

**Table 5:** Correlations between the PRAQ-R/R2 and other anxiety measurement tools (Spearman’s rho coefficient).

Measuring Tool	Scale/Trait	PRAQ-R2 Total Score	PRAQ-R Total Score	PRAQ-R2: FoGB	PRAQ-R: FoGB	PRAQ-R2: WoHC	PRAQ-R2: CoA
VAS	0 to 10 score	0.4 ***	0.34 ***	0.51 ***	0.39 ***	0.25 ***	0.17 *
TIPI	extraversion	−0.24 **	−0.26 ***	−0.17 *	−0.2 **	−0.18 *	−0.22 **
	openness	−0.22 **	−0.23 **	−0.14	−0.13	−0.16 *	−0.16 *
	neuroticism	−0.37 ***	−0.36 ***	−0.27 ***	−0.24 **	−0.29 ***	−0.23 **
	conscientiousness	−0.09	−0.1	−0.04	−0.07	−0.1	−0.07
	agreeableness	−0.06	−0.06	−0.03	−0.03	0.02	−0.16 *
HADS	anxiety	0.37 ***	0.35 ***	0.3 ***	0.22 **	0.33 ***	0.17 *
	depression	0.38 ***	0.37 ***	0.22 **	0.18 *	0.36 ***	0.25 ***

* *p* < 0.05; ** *p* < 0.01; *** *p* < 0.001. PRAQ-R: Pregnancy-Related Anxiety Questionnaire—Revised; FoGB: fear of giving birth; WoHC: worries of bearing a handicapped child; CoA: concern about own appearance; VAS: Visual Analog Score; TIPI: Ten-Item Personality Indicator; HADS: Hospital Anxiety and Depression Score.

## Data Availability

Data available on request due to privacy/ethical restrictions.

## Appendix A

**Table A1:** PRAQ-R/R2 form used in comparative study (original and translated items presented together). Please circle each answer that applies most accurately to your situation. (Zaznacz odpowiedź, która najlepiej pasuje do Twojej sytuacji.) Answer categories: (Kategorie odpowiedzi).

	1 Absolutely Not Relevant *(Stwierdzenie zupełnie mnie nie dotyczy)*	2	3	4	5 Very Relevant *(Stwierdzenie* *w pełni mnie dotyczy)*
1. I am anxious about the delivery. *(Czuję lęk przed porodem)*. *	1	2	3	4	5
2. I am worried about the pain of contractions and the pain during delivery. *(Martwię się bólem w czasie skurczy oraz bólem w czasie porodu).*	1	2	3	4	5
3. I am worried about the fact that I shall not regain my figure after delivery. *(Martwię się, iż nie odzyskam mojej dawnej figury po porodzie).*	1	2	3	4	5
4. I sometimes think that our child will be in poor health or will be prone to illnesses. *(Czasami myślę, że nasze dziecko urodzi się chore lub będzie miało skłonność do chorób).*	1	2	3	4	5
5. I am concerned about my unattractive appearance. *(Uważam, że wyglądam nieatrakcyjnie).*	1	2	3	4	5
6. I am worried about not being able to control myself during labor and fear that I will scream. *(Boję się, że w czasie porodu stracę kontrolę nad sobą i obawiam się, że będę krzyczeć).*	1	2	3	4	5
7. I am worried about my enormous weight gain. *(Martwię się, że znacząco przybieram na wadze).*	1	2	3	4	5
8. I am anxious about the delivery because I have never experienced one before. *(Boję się porodu, ponieważ nigdy jeszcze nie rodziłam).* **	1	2	3	4	5
9. I am afraid the baby will be mentally handicapped or will suffer from brain damage. *(Obawiam się, iż dziecko będzie niepełnosprawne umysłowo lub dozna uszkodzenia mózgu).*	1	2	3	4	5
10. I am afraid our baby will be stillborn or will die during or immediately after delivery. *(Obawiam się, iż nasze dziecko urodzi się martwe lub umrze bezpośrednio po porodzie).*	1	2	3	4	5
11. I am afraid that our baby will suffer from a physical defect or worry that something will be physically wrong with the baby. *(Obawiam się, że dziecko urodzi się z defektem lub będzie fizycznie uszkodzone).*	1	2	3	4	5

* modified universal item for both nulliparous and parous (PRAQ-R2). ** item used only for nulliparous (PRAQ-R)
